# Take it sitting down: the effect of body posture on cortical potentials during free viewing—A mobile EEG recording study

**DOI:** 10.3389/fnins.2024.1492427

**Published:** 2024-11-28

**Authors:** Vicente Soto, John Tyson-Carr, Katerina Kokmotou, Hannah Roberts, Adam Byrne, Danielle Hewitt, Nicholas Fallon, Timo Giesbrecht, Andrej Stancak

**Affiliations:** ^1^Department of Psychology, University of Liverpool, Liverpool, United Kingdom; ^2^School of Psychology, Centre of Social and Cognitive Neuroscience, Universidad Adolfo Ibáñez, Santiago, Chile; ^3^Institute for Risk and Uncertainty, University of Liverpool, Liverpool, United Kingdom; ^4^Unilever Research and Development Port Sunlight Laboratory, Merseyside, United Kingdom

**Keywords:** EEG, eye-movement related potentials, body posture, N170, mobile brain imaging

## Abstract

Brain imaging performed in natural settings is known as mobile brain and body imaging (MoBI). One of the features which distinguishes MoBI and laboratory-based experiments is the body posture. Previous studies pointed to mechanical, autonomic, cortical and cognitive differences between upright stance and sitting or reclining. The purpose of this study was to analyse effects of posture on eye-movement related potentials (EMRP) recorded during free viewing of human faces. A 64-channel wireless EEG was recorded from 14 participants in either standing or reclining postures while they freely viewed pictures of emotional faces displaying fear, anger, sadness, and a neutral emotional state. Eye tracking data was used to insert triggers corresponding to the instant at which the gaze first landed on a face. Spatial filtering of the EEG data was performed using a group independent component analysis (ICA). Grand average EMRPs displayed the post-saccadic lambda component and the face-sensitive N170/vertex positive potential (VPP) complex. The lambda component but not the N170 component was stronger during reclining than upright posture. Emotional expression of faces showed no effects on EMRP components or subjective ratings. Results suggest that posture primarily affects early components of EMRPs recorded using wireless EEG recordings during free viewing of faces. Thus, findings from evoked potential data obtained in seated individuals, e.g., in laboratory experiments, should be interpreted with caution in MoBI experiments with posture affecting primarily the early latency component.

## Introduction

Mobile brain and body imaging (MoBI), entailing multimodal electrophysiological recordings in freely behaving individuals, is a novel type of brain imaging offering the possibilities to understand the brain processes as they spontaneously evolve during natural activities, such as walking or running (Gwin et al., 2010), cycling (Zink et al., 2016), driving (Protzak and Gramann, 2018), talking or free viewing of environments (Roberts et al., 2018; Soto et al., 2018). A wireless electroencephalographic (EEG) recording is an essential component in MoBI experiments to allow for unobstructed continuous recordings during natural cognition.

A feature which often distinguishes MoBI experiments from laboratory-based experiments is the participant’s posture with laboratory experiments being traditionally conducted in seated individuals. Standing is a seemingly simple activity, at times assumed equal to sitting recumbent or laying supine. However, maintaining a standing balanced posture requires the rapid processing and integration of continuous vestibular, somatosensory and visual information (Mergner and Rosemeier, 1998; Nashner, 1976), reviewed in Thibault and Raz (2016).

How body posture affects brain activity has been difficult to study in the past and as such, our scientific understanding is limited. The lack of insight could be partially attributable to the inherent postural requirements of modern brain imaging techniques. The ecological nuances associated with functional magnetic resonance imaging (fMRI) have previously been described (Raz et al., 2005). These revolve around the necessity of supine participants to be restrained and to remain still as stimuli suddenly appear on screen. Positron emission tomography (PET) with modified scanner gantries have been used to study effects of posture on brain activations (Harmon-Jones et al., 2011; Riskind and Gotay, 1982). However, the use of radioactive contrasts as well as the size of the scanner limit the possibility of PET to explore effects of posture on brain processing in freely behaving individuals. EEG recordings classically position sitting subjects alone in dimly lit rooms and viewing monitors, whereas magnetoencephalography (MEG) uses both sitting and supine participants. In all cases, body and head movements are often minimized via chin rests and head braces. The most glaring differences among these imaging modalities is the participant’s posture during recordings and the type of restrictions to body movements. Furthermore, the experimental tasks being performed are, at times, incongruent with the body position that subjects must assume; for instance, performing arithmetic calculations while laying supine (Lipnicki and Byrne, 2008) or performing a driving task inside the scanner environment (Schweizer et al., 2013).

Moreover, modifications to body posture have been shown not only to affect physiological processes such as blood pressure regulation and brain activations, but also behavior and cognition (reviewed in Thibault and Raz, 2016). Standing on both feet compared to supine posture has been shown to increase activation in the right primary visual cortex (Ouchi et al., 1999) and cerebellum (Ouchi et al., 1999, 2001). Upright stances also aid psychomotor performance (Caldwell et al., 2003; Lazarus and Folkman, 1984) and increase anticipatory anxiety during mental arithmetic tasks (Lipnicki and Byrne, 2005). Similarly, a sitting posture has been shown to improve intelligence and augment perception compared to a supine posture (Lundström et al., 2006) and adopting certain postures (i.e., slumping) can reduce emotional responses (Harmon-Jones and Peterson, 2009) and influence rhythmic cortical activity, behavior (Harmon-Jones et al., 2011) and cognitive conflict processing (Sun and Harmon-Jones, 2021). Even small body movements like the adoption of different facial expressions affect emotional judgments and the generation of memories (Laird and Lacasse, 2014). Other studies have shown that leaning direction plays an important role in affect perception (Harrigan and Rosenthal, 1983; Troncoso et al., 2024). These findings suggest that neural processing of emotional stimuli could vary resulting from changes in body positions, alluding to a close-knit relationship between posture and emotional processing. We have previously shown that wirelessly recorded face-sensitive visual evoked components in freely behaving humans differentiated faces displaying four different emotional expressions (Soto et al., 2018). Here, the rationale is to build upon these previous findings to examine the effect of body posture on face processing in the brain. Such effects have been sparsely reported in laboratory studies in the past (Batty and Taylor, 2003; Blau et al., 2007) and a number of laboratory studies failed to show effects of emotional expressions on the face-sensitive N170 component (Chai et al., 2012; Eimer et al., 2003; Hendriks et al., 2007). It is possible however, that an upright stance may increase general arousal and certain emotional processes.

In the present study, we implemented wireless EEG recordings to analyse effects of posture on the early visual evoked component and face-related evoked components. Similar to our previous study (Soto et al., 2018), participants viewed human faces displayed on posters while they were moving or standing freely, and their EEG and eye movements were recorded continuously. In a different session and using the same types of recordings, participants viewed posters with faces in a reclining-supine posture. Faces showed one of four emotional expressions: happy, fearful, disgusted or neutral. We hypothesized, in line with previous data showing increased visual cortex activation in upright stance compared to supine posture (Ouchi et al., 1999), that upright stance would reduce the amplitude of both the early visual EMRP component and the face-sensitive EMRP component due to visual processing demands employed during standing.

## Materials and methods

### Participants

Fifteen healthy volunteers 24.6 ± 3.2 years old (mean ± SD), were recruited for the study by online advertisements and word of mouth. Subjects that wore corrective eye-glasses were excluded from recruitment due to the glare artifact the glass generates in the wearable eye-tracking system. Subjects wearing contact lenses were not excluded from the recruitment as these lenses do not interfere with the eye-tracking system, but were required to wear them for both sessions. One participant from the sample was rejected prior to data processing due to an excessively noisy EEG signal during the reclining session which caused a loss of more than 20% stimuli for one experimental condition. Therefore the final sample was composed of 14 subjects (eight females) with an average age of 24.6 ± 3.3 years. Experimental subjects gave written informed consent prior to taking part in the study in agreement with the ethical approval obtained from the University of Liverpool Research Ethics Committee. Participants received £30 (£15 per session attended) as compensation for their travel expenses and time. All experimental procedures were conducted in two sessions and in accordance with the Declaration of Helsinki.

### Stimuli

**Figure 1:**
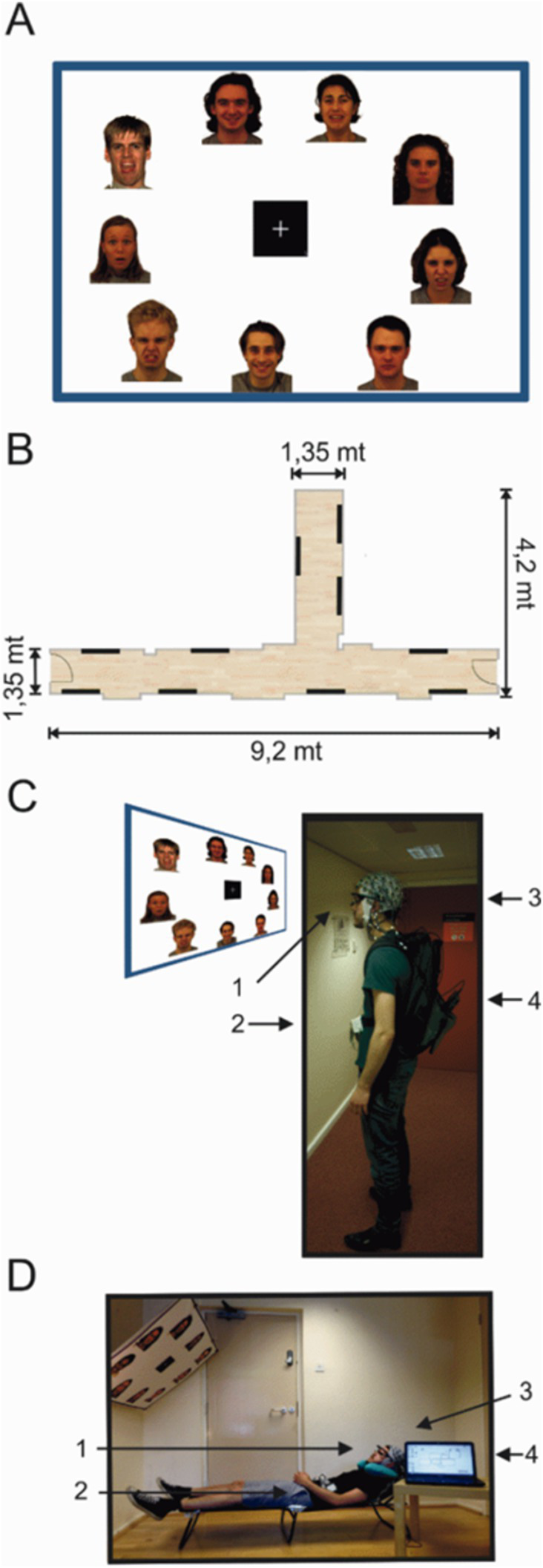
Experimental setup of the mock gallery and reclining sessions. **(A)** Example of one poster panel (120 × 90 cms) presenting face images. **(B)** Schematic illustration of the hallway used to setup the mock gallery. Black lines indicate the position of each poster within the mock gallery. **(C,D)** One subject wearing the full equipment and positioned in the experimental setups for both the standing and reclining sessions. (1) Pupil eye tracker; (2) MOVE wireless EEG transmitter; (3) 64 channel EEG actiCap; (4) Eye tracking PC in the backpack. Face images were selected from the Karolinska Directed Emotional Faces (KDEF; Lundqvist et al., 1998) set and from the NimStim face inventory (Tottenham et al., 2009).

Face images expressing four emotional categories (fearful, disgusted, happy and neutral) were selected from the Karolinska Directed Emotional Faces (KDEF; Lundqvist et al., 1998) set and from the NimStim face inventory (Tottenham et al., 2009). The full stimulus set consisted of 180 emotional human faces, 45 images in each emotional category presented on 20 A0 poster-size sheets (841 × 1,189 mm). A total of 120 images were taken from the KDEF and 60 from the NimStim set. All 180 images used in the experiment were cropped to match in size and quality (300 DPI) using CorelDRAW software. Additionally, all images were matched for temperature and brightness and were always presented as standard portrait shots including the shoulders. A0 size panels were constructed containing nine different image stimuli presented in full color approximately 20 × 25 cm in size, with a white-on-black square fixation cross presented in the centre (14.3 × 14.3 cm) (Figure 1A). Twenty panels contained all emotional face categories pseudo-randomly distributed and maintained similar distances from the fixation cross in the centre. All panels were pasted onto Styrofoam sheets and attached to the walls using adhesive tape creating a mock gallery (Figure 1B).

### EEG recordings

EEG data was continuously recorded using a 64-channel wireless and portable EEG system (Brain Products, GmbH, Münich, Germany). Brain Products MOVE system (Brain Products, GmbH) employs a lightweight signal transmitter which participants carry on a belt (Figures 1C,D). Active shielding Ag/AgCl EEG electrodes were mounted on an electrode cap (actiCAP, Brain Products, GmbH) according to the international 10–20 electrode system, aligned to the midpoint between the anatomical landmarks of the nasion and inion, and left and right preauricular points. Electrode FCz was used as the system ground and electrodes were referenced to Cz. Electrolyte gel was applied to lower electrode-to-skin impedances to under 50 kΩ. EEG recordings were sampled at a rate of 1,000 Hz. EEG average reference was applied and the signals were digitized at 1 kHz with a BrainAmp DC amplifier linked to BrainVision Recorder program version 1.20.0601 running on Windows operating system. Data were filtered online using a 0.1–200 Hz bandpass filter.

### Eye movement recording

A PUPIL (Kassner et al., 2014) binocular eye-tracking system was used to record the locations of the gaze position. PUPIL Capture software v 0.9.6 running on Ubuntu v 14.04.4 was used to generate the recordings. PUPIL eye tracker is an open source, high resolution wearable system (Figure 1C) that provides a lightweight solution for mobile gaze-tracking. Pupil locations were recorded using infra-red cameras sampling at 90 Hz with a resolution of 320 × 240 pixels. The real-world video streams were set at a sampling rate of 60 frames per second with a resolution of 600 × 800 pixels.

To calibrate the gaze locations, a manual marker 3D calibration protocol was used to generate a 9-point grid in the field of view of the participant. Calibration was repeated until gaze positions were accurate everywhere on the blank panel. Small calibration offsets occurred at times due to displacements in the wearable eye-tracker on the subject’s face. These offsets were adjusted offline using the manual gaze correction plug-in on PUPIL Player during manual tabulation of stimulus onset times. Eye-tracking data were processed using PUPIL Player v. 0.9.6 program. The ocular pupils of both eyes were located based on a centre-surround detection algorithm (Świrski et al., 2012). Exported raw gaze data is time-locked to the processing computer’s internal clock, giving millisecond precision to the eye measurements. Additionally, all recorded frames contained an accurate time stamp based on the PC processor real-time clock. Eye-tracking video files were visually inspected and stimuli onsets were manually tabulated. Each stimulus was logged on a picture-by-picture basis with stimulus onset defined as the first instance in which the gaze position landed on an image. The real times corresponding to the tabulated frames were used to import stimulus onset latencies onto the raw EEG data. Eye-tracking and EEG data streams were aligned at the start of each recording using a custom-built light-emitting trigger box, which flashed a light into the eye-tracking camera and synchronously registered a transistor logic pulse in the EEG data recording.

### Procedure

Volunteers attended two testing days, a standing session where stimuli were viewed while upright, and a reclining session where they sat in a recumbent position on a lounge chair. In both sessions, instructions were delivered and equipment was set up in a designated lab space following identical procedures. The order of conditions was counterbalanced across subjects with half of the participants taking part in the reclining task first, and the remaining participants taking part in the standing task first. Experimental sessions were separated by a minimum of 1 week.

### Standing session

Participants were fit with the EEG cap and electrode-to-skin impedances were lowered below 50 kΩ before commencing the task as well as during the break between blocks. The mobile EEG system was connected and wireless signals were visually inspected on a standing participant. Next, eye-tracking glasses were placed on the participant over the EEG cap.

A mobile base unit was assembled using a rolling trolley where the wireless signal receiver, EEG amplifier and recording computer were placed. The base unit was positioned by the experimenter, keeping a distance of no more than 7 m from the participant in order to maintain optimal signal quality. The eye-trackers were calibrated on a standing participant against a blank white panel at a distance of 1 m from the centre of the panel to match the viewing conditions during the mock gallery. Gaze-tracking was optimized using a 3D calibration routine with manual markers. The laptop registering the eye-tracking was placed in a backpack and carried by the participant for the duration of the task (Figures 1C,D). EEG cables running from the electrodes to the lightweight transmitter were also placed in the backpack to reduce cable sway artifacts (Gramann et al., 2010; Gwin et al., 2010).

Two hallways within the Eleanor Rathbone Building of the University of Liverpool were used to create a naturalistic picture gallery where the standing session took place (Figure 1B). Participants were free to view images in any order and to navigate the gallery however they chose. All subjects were asked to stand still and view each image for a minimum of a few seconds before moving onto the next image. To do so, subjects were instructed to stand at a set distance of 1 m and remain still. They first looked at the centre fixation cross before viewing each image, and were requested to return their gaze to the fixation cross before moving to the next picture. Participants only continued onto a subsequent panel after viewing all images. To maintain task engagement and to avoid drowsiness, participants were asked to select their most and least preferred face from each panel and to mark the selected pictures on a small paper-printed version of each panel. The full experiment consisted of two blocks in the mock gallery. In each block, subjects viewed 10 panels with nine images on each one. In total, participants viewed 180 different images in the experiment. On average, each of the two gallery blocks lasted approximately 15 min.

### Reclining session

The wireless EEG was placed on the participant in the same manner as in the standing session. For the reclining task, the eye-trackers were calibrated on a blank white panel with the participant lying on the lounge chair. The blank panel was hung at a distance of approximately 1 m with 140° angle to match viewing conditions across both sessions. The inclination angle of the lounge chair was lifted to approximately 40° (Figure 1D) bringing the fixation cross in the centre to the participant’s eyeline. In this session, subjects laid on a lounge chair that provided full body support as high as the neck. A foam neck support was used to prevent electrodes on the back of the scalp smearing against the chair and provided head support. An identical 3D calibration routine using manual markers was used to optimize the gaze-tracking for the reclined condition. The eye-tracking computer was placed on the same mobile base unit where the EEG recording computer rested in this session.

Participants were instructed to view the images displayed on each panel. The experimenters hung each panel as they were completed by the participants. Subjects were instructed to relax and view each face for a couple of seconds before returning to the fixation cross and moving onto the next image. As in the standing session, no restrictions were placed on eye-movements or blinks. The same selection task was used here to maintain engagement and avoid drowsiness. Participants viewed the same 180 different images in the experiment divided into two blocks with breaks between them. Each block in the reclined session also lasted approximately 15 min.

In both sessions, electrode impedances and gaze tracking calibration were checked in the break between blocks and corrected if required. Once the viewing task was completed, the EEG cap and the eye-tracking glasses were removed. Participants were then required to rate how much they liked and if they would approach the images that had been previously seen. Ratings were performed using three visual analogue scales (VAS) sized 10 cm and anchored on each extreme. The scales evaluated the general likability of each face image as well as the level of arousal and emotional valence. Subjects were instructed to use the middle of the scale for expressions deemed neutral. (i.e., “*0: Do not like*” up to “*100*: *Like very much*”; “*0: Unarousing*” up to “*100: Very Arousing*” *and* “*0: Very negative*” up to “*100: Very positive*”). Pictures and rating scales were presented on an LCD screen using Cogent program v. 1.32 (Welcome Department of Imaging Neuroscience, United Kingdom) running on MATLAB v. R2014a (MathWorks, Inc., USA). Participants rated the same faces after each session.

## EEG analysis

### Eye movement related potentials

EEG data were pre-processed using the Brain Electrical Source Analysis program (BESA v.6.0, MEGIS Software GmbH, Munich, Germany). Data were initially referenced using the common averaging method (Lehmann, 1987) and digital filters were applied to remove frequencies lower than 0.5 Hz and higher than 35 Hz from the EEG data to minimize electromagnetic interference (EMI) from electrical appliances. Ocular artifacts including blinks as well as vertical and horizontal eye-movement artifacts were removed using pattern matching averaged artifact topographies (Berg and Scherg, 1994; Ille et al., 2002). This method is based on a principal component analysis and creating individualized artifact topographies to maximally match and resolve eye-movement artifacts in EEG data. Additionally, EEG data was visually inspected and corrected for the presence of remaining artifacts. Trials were excluded if artifacts were present in either eye-tracking or EEG data. If participants skipped an image, failed to fixate, or gaze tracking was lost during fixation, the trial was discarded from any further analysis. To ensure consistency across our conditions, subjects with more than 20% rejected epochs for one experimental condition were eliminated from the final analysis.

Individual stimulus onset times were detected by visual inspection of the eye-tracking data. For all stimuli the first instance where a participant’s gaze made contact with a stimulus image was registered as visual onset time for event marking. Event markers were then inserted into EEG data by synchronizing the time axes of the EEG and eye-tracking system in MATLAB (The MathWorks Inc., 2018). EEG data were epoched to range from −200 ms to 600 ms relative to the first contact of the gaze with any part of a picture in each of 180 pictures. This time point effectively corresponded to part of the saccade which brought the gaze onto a particular face or object in a picture. Artifact-corrected EEG signals were then exported into EEGLAB v.14.1.1,^1^ an open-source environment for processing EEG data (Delorme and Makeig, 2003). During averaging, the mean EEG activity in the baseline interval ranging from −200 ms to −100 ms was removed from each data point as it represented a more stable baseline relative to the −100 ms to 0 ms in free viewing conditions. Post-saccadic EMRPs were computed from all trials falling into eight different conditions including postural conditions (reclining and standing) and emotional facial expressions (afraid, disgusted, neutral and happy). The sampling rate of the eye-tracking system was previously calculated offline (average sampling rate: 41.1 Hz). The sampling rate was chosen based on 15-min pilot experiments to secure a continuous stream of eye-tracking data which was often discontinuous at higher sampling rates. However, due to the relatively low sampling rate, further analysis of the eye movements was not performed and eye-tracking data was used exclusively to detect stimuli onset times. EEG epochs were generated as cuts into the world video scene in a similar manner as the method described in our previous experiment (Soto et al., 2018). Artifact-corrected EEG data was then exported to EEGLAB for group-level analysis.

### Data analysis and independent component clustering

Individual subject data was entered into the EEGLAB STUDY structure (Delorme et al., 2011). Independent component analysis (ICA) was performed within EEGLAB for all subjects using an extended Infomax ICA algorithm to parse EEG by defining maximally statistically independent components (IC) (Dimigen, 2020; Makeig et al., 1996) using the default training parameters on EEGLAB. This method allows an accurate detection and removal of extra-cerebral sources of noise. ICA was performed on concatenated epochs for each subject individually. Event-related potentials (ERPs) and IC scalp maps were then computed across experimental conditions and used to construct a pre-clustering array to group ICs into 9 clusters. The DIPFIT2 routine from EEGLAB was used to model each IC as an equivalent current dipole by using a standardized boundary element (Oostenveld and Oostendorp, 2002). If the residual variance of the single-dipole exceeded 30%, or if the location landed outside the head, the IC was removed prior to clustering. The clustering of ICs was performed across all 14 subjects based on similarities in scalp topographies, equivalent dipole locations and ERP waveforms by means of EEGLAB’s *k*-means clustering routine (Lee et al., 2015). Three clusters of interest were identified and selected based on IC activity, average dipole locations and the presence of ICs in at least nine subjects. Furthermore, the two peak features in the global field power were used to identify component clusters for analysis. Cluster 2 presented 33 ICs across 13 subjects and accounted for the N170 component. Cluster 8 (16 ICs), clustered from nine subjects, presented a small peak in activity at around 90 ms and accounted for the vertex positive potential (VPP) peaking in medial scalp locations and a larger secondary peak at around 170 ms. Cluster 6 was composed of 18 ICs present in 10 subjects and represented the major contributor to the lambda component (Kazai and Yagi, 2003; Vigon et al., 2002). Statistical testing was performed on the grand averaged IC cluster activity across all experimental conditions for these three components.

### Statistical analysis

The source waveforms representing the averaged clustered IC activity in each of the fitted dipoles were analysed using a 2 × 4 ANOVA for repeated measures ANOVA (Reclining vs. standing, four emotional face conditions). Specifically, a permutation-based repeated-measures ANOVA utilizing 5,000 permutations was employed (Maris and Oostenveld, 2007). Statistical testing was performed on the averaged source activity across the intervals of interest in SPSS v.22 (IBM Corp., NY, USA). We focused the analysis on 20 ms time intervals (−10 to 10 ms) relative to the peak in IC activity for each selected cluster. Post-hoc paired *t*-tests were performed when necessary and considered significant at *p* ≤ 0.05 and Bonferroni corrections for multiple comparisons were always used.

## Results

### Face ratings

**Figure 2:**
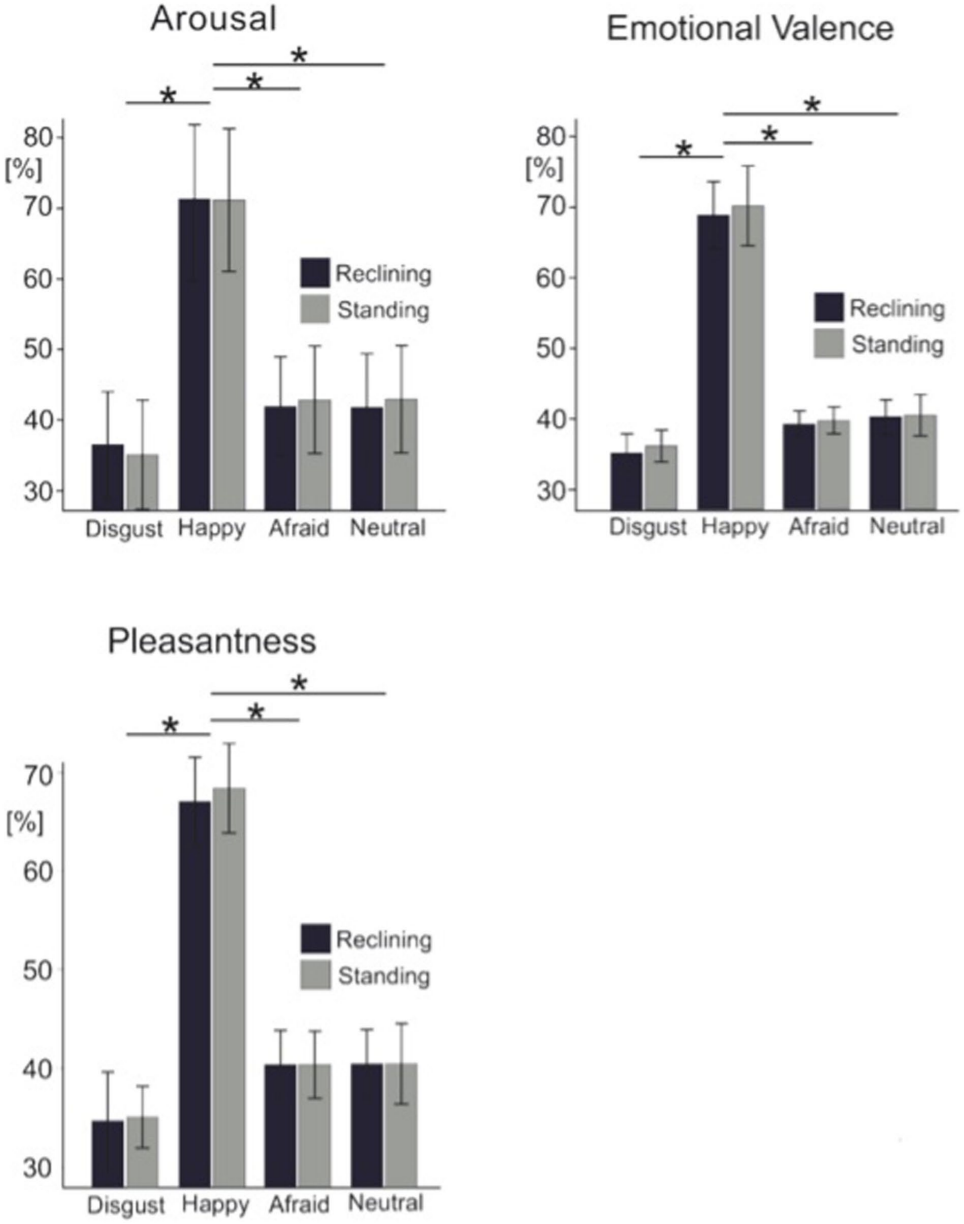
Grand average ratings for each visual analogue scale. Light color bars represent the standing condition while dark color bars represent the reclining condition. Error bars represent 1 standard deviation. Asterisks represent significance at *p* < 0.05.

Figure 2 shows the average rating scores for each visual analogue scale across all four experimental conditions. To test the effects of body posture on the overt rating behavior, each of the three individual visual analogue scale ratings were submitted to separate 2 × 4 ANOVAs for repeated measures. Across all rating scales, we found no effects of posture observed on overt rating behavior between standing and reclining conditions [arousal scale: *F*_(1, 14)_ = 0.009; *p* = 0.927; emotion detection scale: *F*_(1, 14)_ = 1.112; *p* = 0.31; and pleasantness scale: *F*_(1, 14)_ = 0.251; *p* = 0.624]. A significant effect of emotional condition on arousal level was also found in this analysis [*F*_(3, 42)_ = 100.62, *p* < 0.001] as well as for the emotion [*F*_(3, 42)_ = 69.008, *p* < 0.001] and pleasantness scale [*F*_(3, 42)_ = 84.883, *p* < 0.001].

Pairwise *t*-tests were used to test differences among all pairs of emotional categories. Paired comparisons confirmed that arousal ratings were significantly different across emotional expressions specifically; disgusted expressions rated as less arousing than afraid facial expressions [*t*(14) = −6.1; *p* = 0.003] and more arousing than neutral faces [*t*(14) = −2.9; *p* = 0.012]. Unexpectedly, afraid and neutral facial expressions were rated as equally arousing by our participants [*t*(14) = 0.104; *p* = 0.92]. Similarly, the emotional scale ratings showed happy faces (80.7 ± 7.8, mean ± SD) were consistently rated as highly positive while disgusted (35.2 ± 6.6), and afraid (39.1 ± 5.7) were rated on the negative end of the scale. Neutral facial expressions, however, were perceived to be slightly negative (41.1 ± 6.8). As expected, this effect was additionally encountered in the pleasantness ratings where faces reflecting happy emotional expressions were perceived as more pleasant than disgusted [*t*(14) = −12.6, *p* < 0.001], afraid [*t*(14) = 14.1; *p* < 0.001] or neutral faces [*t*(14) = 10.4, *p* < 0.001]. Again, neutral faces were rated equally pleasant as disgusted faces [*t*(14) = −2.1; *p* > 0.05] and afraid expressions [*t*(14) = 0.012, *p* > 0.05] irrespective of the postural position.

### Eye movement related potentials

**Figure 3:**
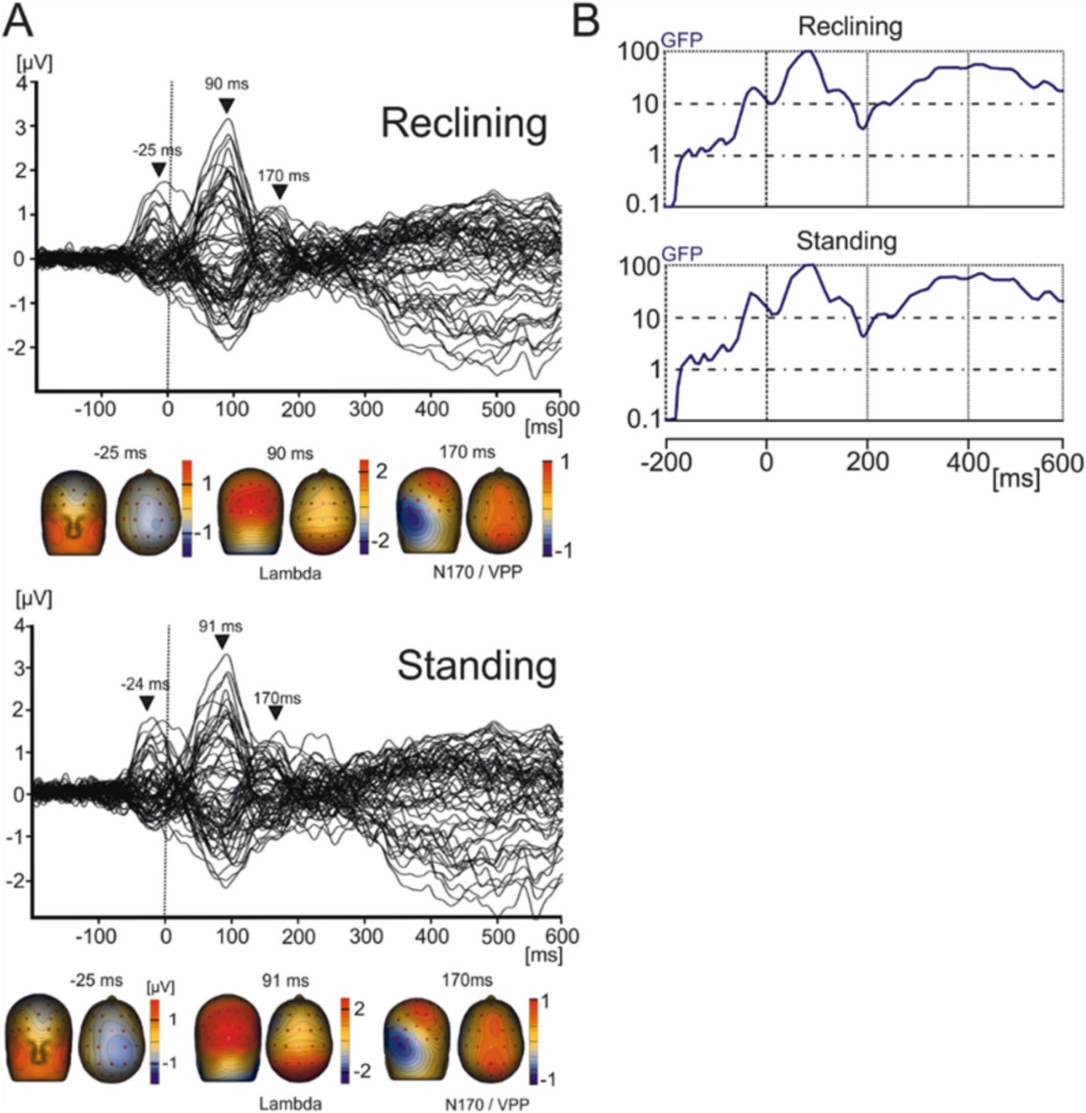
Grand average EMRP for the reclining and standing conditions. **(A)** Butterfly plots of the average EMRP waveforms between 200 ms and 600 ms. Peak latencies and scalp topographies overlaid on the volume rendering of the human head at three latency points in reclining (−25, 90, and 170 ms) and standing conditions (−24, 91, and 170 ms). EMRP components are highlighted with arrows. **(B)** Global field power of the EMRP for the reclining and standing session.

EMRPs were constructed from −200 ms to 600 ms relative to the first instance of contact of the subject’s gaze with an image (Figure 3). Given participants remained still to view images the EEG data showed minimal neck muscle or head movement artifacts, which have been shown to affect EEG data during walking or running (Gwin et al., 2010).

### Independent component clusters

IC clusters were selected on the basis of their maximum explained variance being >70% and that clusters were composed of IC from a minimum of seven subjects. Additionally, the spatial–temporal characteristics of the scalp maps and dipole locations were used to select ICs.

**Figure 4:**
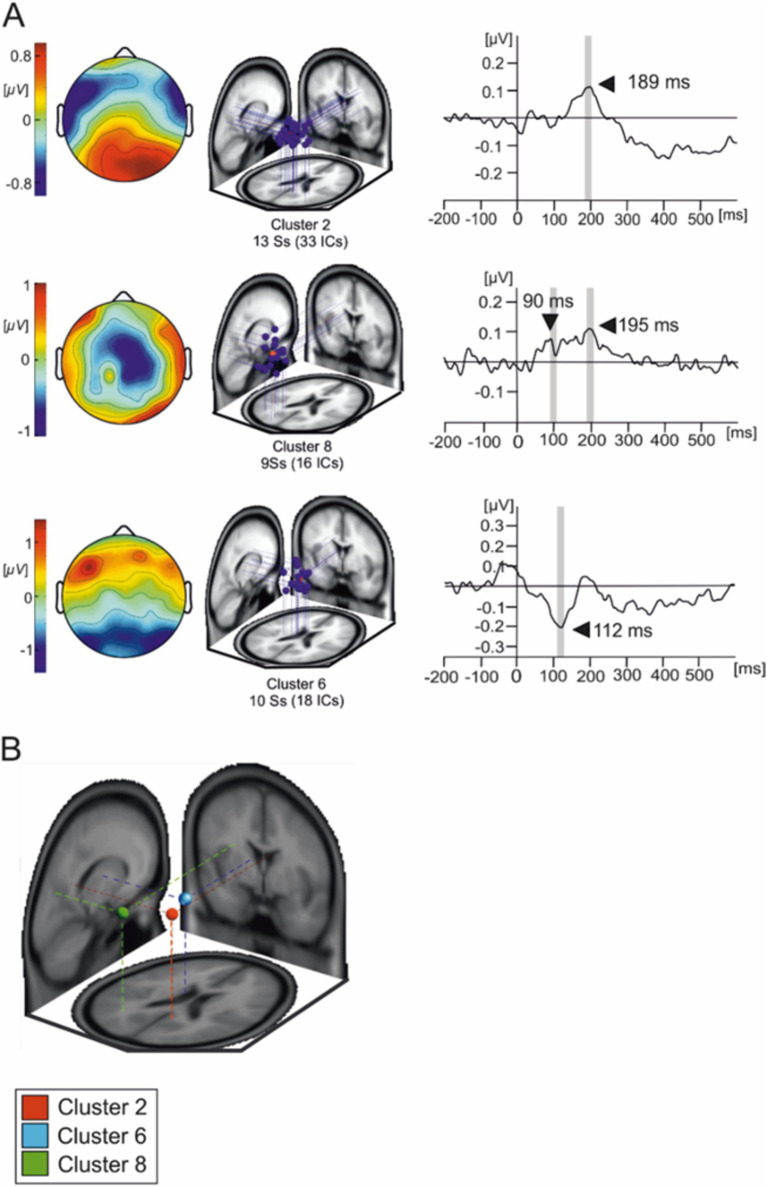
Independent components composing the three IC clusters of interest. **(A)** Overlaid scalp plots of each cluster of interest (IC cluster 1, IC cluster 8, and IC cluster 6). Scalp plot polarities presented here are arbitrary. The shaded time interval where statistical testing was performed. **(B)** Average IC cluster locations and projection lines on a three-slice MRI template (red sphere represents the averaged dipole location for cluster 2, blue sphere represents the averaged dipole location for cluster 6 and green represents the averaged dipole location for cluster 8).

Three IC clusters were selected based on the peak in their averaged waveforms and scalp distributions (Figure 4). The mean IC dipole for each IC cluster of interest was located in, or near to the posterior cingulate cortex (cluster 2), the left thalamus (cluster 6) and the left angular gyrus (cluster 8). The average waveform activations of each of these IC clusters were statistically tested using a 2 × 4 (body posture × emotional condition) ANOVA for repeated measures.

Cluster 2 presents as a tightly grouped cluster of 33 ICs in occipito-temporal portions of the brain present in 13 subjects. However, we must interpret averaged cluster localizations findings with caution given that dipole localization in EEG, while informative, lacks the spatial resolution to definitively determine the precise neural generators of the observed activity.

**Figure 5:**
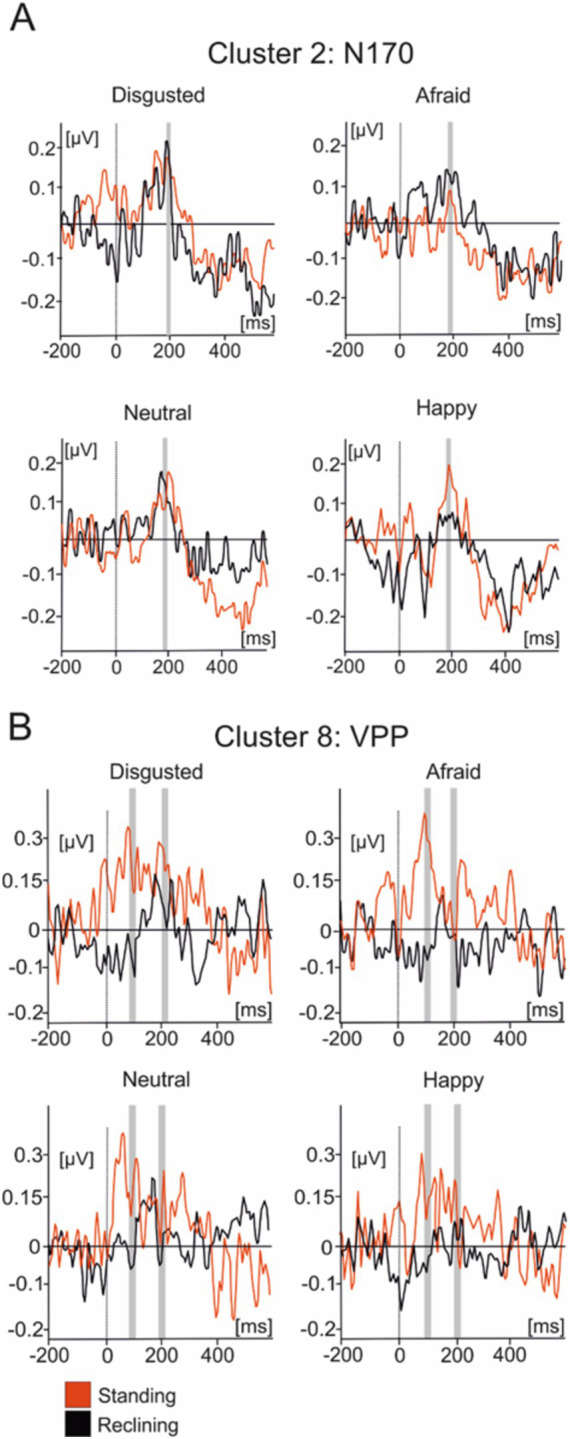
Average IC cluster activations for the N170 and VPP components. **(A)** IC activity accounting for the N170 between −200 ms and 600 ms. **(B)** IC activity accounting for the VPP between −200 ms and 600 ms. Red lines represent the standing condition and black lines represent the reclining condition. Shaded areas represent the time intervals where statistical testing was performed.

Figure 5A illustrates the averaged IC activity across experimental conditions as well as the location of individual ICs composing clusters (Figure 4B). Due to the peak latency and estimated average location of the cluster, we have associated it to the N170 face-sensitive component. The average IC dipole location of this cluster was estimated to be located medially in the posterior cingulate cortex behind the splenium of the corpus callosum (Talairach coordinates x, y, and z: 9, −48, 23; Brodmann Area 23). The mean IC dipole accounted for 86.72% of the total explained variance (13.28% RV ± 8.8; x̄± SD). Across the different experimental conditions, cluster 2 showed peaks in mean activity occurring between 150 ms and 230 ms with an average peak at 189 ms. The ANOVA performed over the 180 ms to 200 ms time interval reflected no effects of body position on cluster 2 [*F*_(1, 13)_ = 0.033; *p* > 0.05]. No effects of emotional condition were present in the data [*F*_(3, 39)_ = 0.776; *p* > 0.05] nor was there an observable interaction effect [*F*_(3, 39)_ = 0.235; *p* > 0.05].

Cluster 8 was composed of 16 components present in nine subjects. The scalp distributions presented over the occipito-parietal regions of the scalp with a first small deflection occurring at roughly 100 ms and a main, larger peak at a latency of about 200 ms (Figure 5B). This cluster modeled the VPP which, given their co-occurrence and shared generators, has been theorized to represent the same brain mechanism as the N170 component (Joyce and Rossion, 2005). The averaged dipole explained 81.17% of the residual variance (19.83% ± 5.94; x̄ ± SD) and was located to the left angular gyrus (Brodmann Area 39, Talairach coordinates: −26, −62, 17). Statistical comparisons were performed in the time interval between 90 ms and 110 ms as well as between 190 ms and 210 ms. Within the first time interval, no significant effect of posture on evoked source activity of cluster 8 [*F*_(1, 13)_ = 2.603, *p* > 0.1] was found in the latency period surrounding the peak. Additionally, no emotional effect [*F*_(3, 39)_ = 0.198, *p* > 0.5] nor was there any effect of interactions between posture and emotional categories [*F*_(3, 39)_ = 0.99, *p* > 0.1]. A similar pattern of results are in the interval ranging from 190 ms to 210 ms with no effects of posture [*F*_(1, 14)_ = 0.007, *p* > 0.5] or emotional category [*F*_(3, 39)_ = 1.426, *p* > 0.1] nor were there any interaction effects present [*F*_(3, 39)_ = 1.677, *p* > 0.05] in the data.

**Figure 6:**
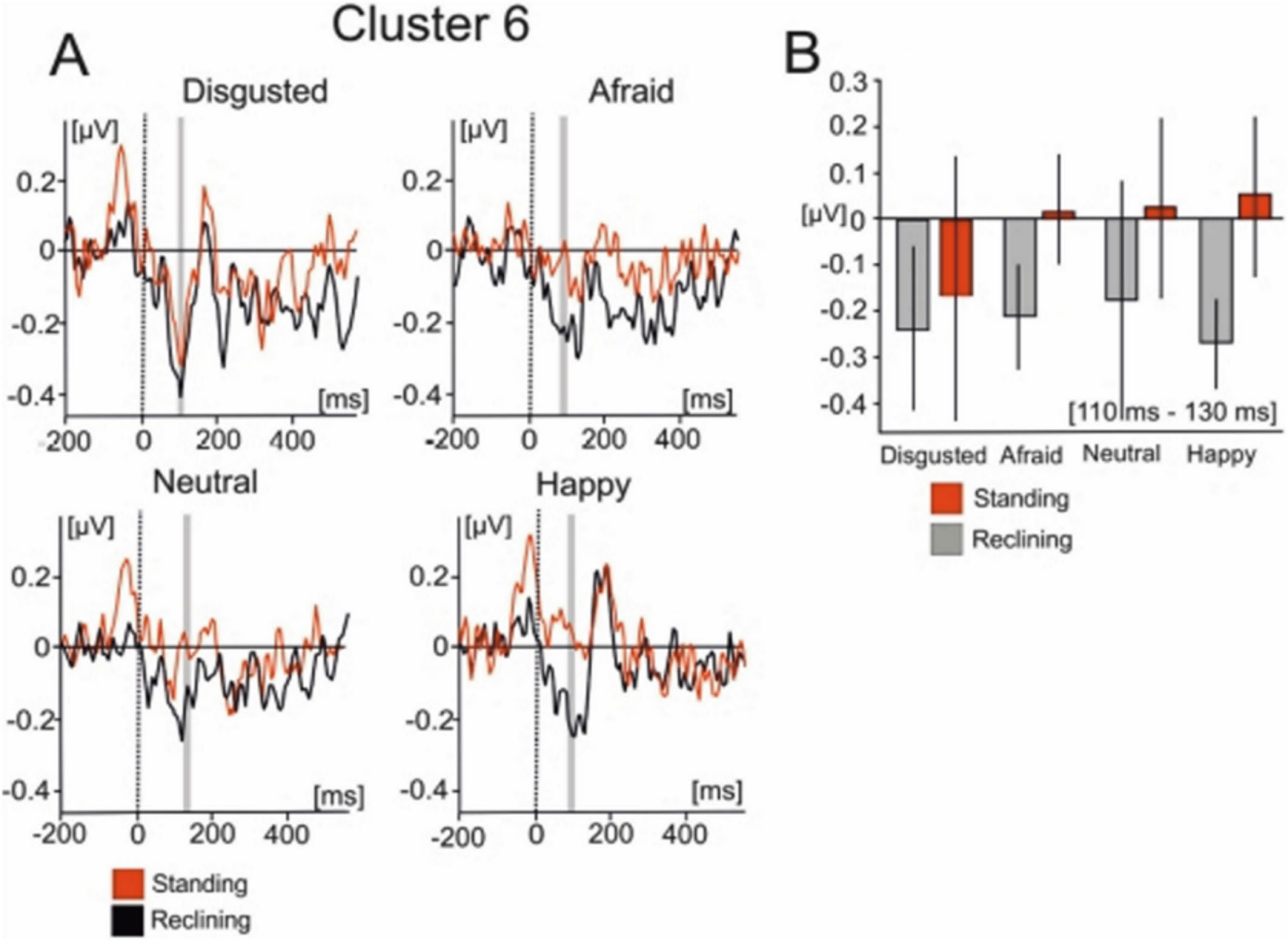
Mean IC cluster activation for cluster 6. **(A)** IC activity between −200 ms to 600 ms for each emotional condition. Red lines represent the standing condition and black lines represent the reclining condition. **(B)** Bar graphs represent the averaged IC activity over the shaded area between 110 and 130 ms.

Cluster 6 was comprised of 18 ICs from 10 of the subjects which were active within similar latencies as the lambda potential (Figure 6). The scalp distribution of the component shows a clear occipital distribution. The averaged IC dipole was fit to the left thalamus (Talairach coordinates −10, −6, 9) and the mean dipole accounted for 84.6% of the explained variance (16.4% ± 9.63). Cluster 6 activations showed significant effects of body posture on IC activity [*F*_(1, 13)_ = 6.424; *p* < 0.05]. The averaged IC activity was larger when subjects reclined (−0.15 ± 0.621 μV) relative to when they stood (−0.085 ± 0.415 μV). There was no effect of emotion on the mean IC cluster activity [*F*_(3, 39)_ = 0.475; *p* > 0.05] nor was an interaction effect found in this interval [*F*_(3, 39)_ = 0.694; *p* > 0.05].

## Discussion

The present study analysed EMRPs recorded wirelessly during viewing face photographs in an upright stance and a reclining posture. Results show that upright body postures reduced the lambda component, but no effect of body posture was found in the N170 component amplitude. None of the EMRP components differentiated emotional expressions of faces.

The lambda response has been identified primarily as a visual response during natural viewing (Thickbroom et al., 1991) and is regarded as the analogue of the P100 component elicited in laboratory-based environments (Kazai and Yagi, 2003). The lambda potential is similarly elicited by the afferent inflow beginning at onset of the visual stimulus (Yagi, 1981) and is thought to represent rapid indexing of visual stimuli and, like the P100 component, is also modulated by low-level visual features (Pourtois et al., 2005) and saccade size (Dimigen et al., 2011). Previous research has shown that leaning forward in a sitting position enhances the P100 component in response to sexually erotic images (Price et al., 2012), and shortens reaction times and increases left frontal cortical activity in response to appetitive food cues when compared to recumbent sitting positions (Harmon-Jones et al., 2011).

Our finding of a reduced lambda component during standing compared to reclining can be interpreted in the light of previous study demonstrating an increased visual cortex activation during standing compared to sitting (Ouchi et al., 1999). Such increased activation associated with standing might reduce the capacity of the visual system to process certain features of the environment which are not related to locomotion or maintaining an upright stance. It is therefore possible that reclining posture favored focused attention during free viewing of faces. It has been suggested that the P100 component also reflects discrete attentional mechanisms in which the enhancement of the P100 component would be invoked by the suppression of unattended stimuli (Clark and Hillyard, 1996). The focusing of visual attention has consistently yielded enhanced P1 amplitudes in laboratory-based testing in the past (Di Russo and Spinelli, 1999; Hillyard and Anllo-Vento, 1998). The P100 enhancement effect has been explained by the recruitment of additional extrastriate neurons when attention is focused on the image (Luck et al., 2000). In general, it is well established that visual evoked potentials are larger during focused attention (Van Voorhis and Hillyard, 1977) and previous ERP research has shown color, shapes and motion can modulate visual ERPs starting at about 100 ms (Anllo-Vento et al., 1998; Torriente et al., 1999). It is therefore likely that an increased lambda component in reclining posture is related to an increased capacity of the visual cortex to engage in a visual task which may be reduced during standing due to the increased posture-related activation of the visual cortex.

Another factor potentially contributing to an increased lambda component in reclining posture compared to standing may refer to the amount of low-frequency theta and alpha oscillations in different postures. Upright stance has been associated with increased high-frequency gamma oscillations but reduced frequency delta, theta and alpha oscillations (Chang et al., 2011; Thibault et al., 2014). The reduction of theta-and alpha-band oscillations during upright stance is relevant for interpretation of decreased lambda EMRP component during standing because the phase-locked evoked potentials can be viewed as a phase reset of underlying spontaneous oscillations (Brandt, 1997; Hanslmayr et al., 2007; Mazaheri and Picton, 2005). This aligns with previous studies showing supine postures reduce frontal alpha activity during rest (Price et al., 2012) and viewing of emotional images (Harmon-Jones and Peterson, 2009) Previous research into the energy distribution in the frequency domain has shown that the P100 component corresponds to increases in power in a wide range of frequency bands, primarily within the theta-and alpha-bands (Quiroga et al., 2001). This might not be the case for later cortical components, however. Recent ERP research has shown a supine body posture would reduce the amplitude of the error-related negativity (ERN) component due to a lowering in approach motivation (Sun and Harmon-Jones, 2021).

Our finding of a decreased lambda component during standing compared to reclining can, in an unknown manner, also be related to mechanical factors such as displacement of the brain inside the skull and changes in thickness of the cerebral spinal fluid surrounding the brain (Thibault and Raz, 2016). Changing from a prone to supine position has shown to generate decreases in cerebrospinal fluid thickness of approximately 30% which can in turn strongly influence occipital EEG signals (Rice et al., 2013). Further studies must address the potential effects of different propagation of electrical currents from the brain in different body postures. However, the lack of posture effects on the face-sensitive EMRP in the present study suggests that the mechanical and current propagation factors played a small role since all EMRP components would likely be affected.

To conclude, we utilized wireless EEG recordings to demonstrate that posture selectively reduces the amplitude of EMRPs. Specifically, upright stances reduced the amplitude of early lambda but not face-sensitive N170-like EMRPs, compared to reclining postures. As outlined, these findings are consistent with previous data showing increased visual cortex activation and increased attentional allocation during standing compared to sitting. The finding of a reduced lambda component in upright stance is important for the evaluation of subsequent MoBI studies involving visual processing in freely moving or standing individuals in natural settings, as early visual evoked components may be attenuated compared to laboratory-based recordings.

Finally, since participants were free to move naturally, their head movements were neither restricted nor controlled. This introduces the possibility that subtle differences in head position and movement across experimental conditions may have influenced the EEG signals. Such challenges are common in mobile EEG research and warrant further attention. Currently, only one project, ICMOBI (2024), is developing analysis tools to automatically distinguish brain and non-brain components in mobile EEG data.^2^ Future studies employing mobile EEG and eye-tracking will benefit from standardized preprocessing pipelines and automated artifact detection methods. While active electrodes significantly reduce the impact of electromagnetic interference (EMI), further advances in electrode shielding and robustness will also be necessary. Moreover, it is important to acknowledge the limitations of using EEG for dipole localization. Future research should adopt multimodal imaging approaches, such as mobile fNIRS-EEG studies, to more accurately resolve the neural sources of these components in the real world.

## Data Availability

The raw data supporting the conclusions of this article will be made available by the authors, without undue reservation.

## Footnotes

^1^ http://www.sccn.ucsd.edu/eeglab/

^2^ https://www.icmobi.org/

## References

[ref1] Anllo-Vento L. Luck S. J. Hillyard S. A. (1998). Spatio-temporal dynamics of attention to color: evidence from human electrophysiology. Hum. Brain Mapp. 6, 216–238. doi: 10.1002/(SICI)1097-0193(1998)6:4<216::AID-HBM3>3.0.CO;2-6, PMID: 9704262 PMC6873357

[ref2] Batty M. Taylor M. J. (2003). Early processing of the six basic facial emotional expressions. Cogn. Brain Res. 17, 613–620. doi: 10.1016/S0926-6410(03)00174-5 14561449

[ref3] Berg P. Scherg M. (1994). A multiple source approach to the correction of eye artifacts. Electroencephalogr. Clin. Neurophysiol. 90, 229–241. doi: 10.1016/0013-4694(94)90094-9, PMID: 7511504

[ref4] Blau V. C. Maurer U. Tottenham N. McCandliss B. D. (2007). The face-specific N170 component is modulated by emotional facial expression. BBF 3:7. doi: 10.1186/1744-9081-3-7, PMID: 17244356 PMC1794418

[ref5] Brandt M. E. (1997). Visual and auditory evoked phase resetting of the alpha EEG. Int. J. Psychophysiol. 26, 285–298. doi: 10.1016/S0167-8760(97)00771-X, PMID: 9203010

[ref6] Caldwell J. A. Prazinko B. Caldwell J. L. (2003). Body posture affects electroencephalographic activity and psychomotor vigilance task performance in sleep-deprived subjects. Clin. Neurophysiol. 114, 23–31. doi: 10.1016/S1388-2457(02)00283-3, PMID: 12495760

[ref7] Chai H. Chen W. Z. Zhu J. Xu Y. Lou L. Yang T.. (2012). Processing of facial expressions of emotions in healthy volunteers: an exploration with event-related potentials and personality traits. Neurophysiol. Clin. 42, 369–375. doi: 10.1016/j.neucli.2012.04.087, PMID: 23181967

[ref8] Chang L. J. Lin J. F. Lin C. F. Wu K. T. Wang Y. M. Kuo C. D. (2011). Effect of body position on bilateral EEG alterations and their relationship with autonomic nervous modulation in normal subjects. Neurosci. Lett. 490, 96–100. doi: 10.1016/j.neulet.2010.12.034, PMID: 21182897

[ref9] Clark V. P. Hillyard S. A. (1996). Spatial selective attention affects early extrastriate but not striate components of the visual evoked potential. J. Cogn. Neurosci. 8, 387–402. doi: 10.1162/jocn.1996.8.5.387, PMID: 23961943

[ref10] Delorme A. Makeig S. (2003). EEGLAB: an open source toolbox for analysis of single-trial EEG dynamics including independent component analysis. J. Neurosci. Methods 134, 9–21. doi: 10.1016/j.jneumeth.2003.10.009, PMID: 15102499

[ref11] Delorme A. Mullen T. Kothe C. Akalin Acar Z. Bigdely-Shamlo N. Vankov A.. (2011). EEGLAB, SIFT, NFT, BCILAB, and ERICA: new tools for advanced EEG processing. Comput. Intell. Neurosci. 2011, 1–12. doi: 10.1155/2011/130714, PMID: 21687590 PMC3114412

[ref12] Di Russo F. Spinelli D. (1999). Electrophysiological evidence for an early attentional mechanism in visual processing in humans. Vis. Res. 39, 2975–2985. doi: 10.1016/S0042-6989(99)00031-0, PMID: 10664797

[ref13] Dimigen O. (2020). Optimizing the ICA-based removal of ocular EEG artifacts from free viewing experiments. Neuro Image 207:116117. doi: 10.1016/j.neuroimage.2019.116117 31689537

[ref14] Dimigen O. Sommer W. Hohlfeld A. Jacobs A. M. Kliegl R. (2011). Coregistration of eye movements and EEG in natural reading: analyses and review. J. Exp. Psychol. Gen. 140, 552–572. doi: 10.1037/a0023885, PMID: 21744985

[ref15] Eimer M. Holmes A. McGlone F. P. (2003). The role of spatial attention in the processing of facial expression: an ERP study of rapid brain responses to six basic emotions. Cogn. Affect. Behav. Neurosci. 3, 97–110. doi: 10.3758/CABN.3.2.97, PMID: 12943325

[ref16] Gramann K. Gwin J. T. Bigdely-Shamlo N. Ferris D. P. Makeig S. (2010). Visual evoked responses during standing and walking. Front. Hum. Neurosci. 4:202. doi: 10.3389/fnhum.2010.00202, PMID: 21267424 PMC3024562

[ref17] Gwin J. T. Gramann K. Makeig S. Ferris D. P. (2010). Removal of movement artifact from high-density EEG recorded during walking and running. J. Neurophysiol. 103, 3526–3534. doi: 10.1152/jn.00105.2010, PMID: 20410364 PMC3774587

[ref18] Hanslmayr S. Klimesch W. Sauseng P. Gruber W. Doppelmayr M. Freunberger R.. (2007). Alpha phase reset contributes to the generation of ERPs. Cereb. Cortex 17, 1–8. doi: 10.1093/cercor/bhj129, PMID: 16452640

[ref19] Harmon-Jones E. Gable P. A. Price T. F. (2011). Leaning embodies desire: evidence that leaning forward increases relative left frontal cortical activation to appetitive stimuli. Biol. Psychol. 87, 311–313. doi: 10.1016/j.biopsycho.2011.03.009, PMID: 21440032

[ref20] Harmon-Jones E. Peterson C. K. (2009). Supine body position reduces neural response to anger evocation: short report. Psychol. Sci. 20, 1209–1210. doi: 10.1111/j.1467-9280.2009.02416.x, PMID: 19656336

[ref21] Harrigan J. A. Rosenthal R. (1983). Physicians’ head and body positions as determinants of perceived rapport. J. Appl. Soc. Psychol. 13, 496–509. doi: 10.1111/j.1559-1816.1983.tb02332.x

[ref22] Hendriks M. C. P. Van Boxtel G. J. M. Vingerhoets A. J. F. M. (2007). An event-related potential study on the early processing of crying faces. Neuro Rep. 18, 631–634. doi: 10.1097/WNR.0b013e3280bad8c7 17426588

[ref23] Hillyard S. A. Anllo-Vento L. (1998). Event-related brain potentials in the study of visual selective attention. Proc. Natl. Acad. Sci. 95, 781–787. doi: 10.1073/pnas.95.3.781, PMID: 9448241 PMC33798

[ref24] ICMOBI. (2024). Available at: https://www.icmobi.org/ (Accessed April 14, 2024).

[ref25] Ille N. Berg P. Scherg M. (2002). Artifact correction of the ongoing EEG using spatial filters based on artifact and brain signal topographies. J. Clin. Neurophysiol. Off. Pub. Am. Electroencephalographic Soc. 19, 113–124. doi: 10.1097/00004691-200203000-00002, PMID: 11997722

[ref26] Joyce C. Rossion B. (2005). The face-sensitive N170 and VPP components manifest the same brain processes: the effect of reference electrode site. Clin. Neurophysiol. 116, 2613–2631. doi: 10.1016/j.clinph.2005.07.005, PMID: 16214404

[ref27] Kassner M. Patera W. Bulling A. (2014). “Pupil: an open source platform for pervasive eye tracking and Mobile gaze-based interaction,” in *Proceedings of the 2014 ACM International Joint Conference on Pervasive and Ubiquitous Computing Adjunct Publication-UbiComp’14 Adjunct*, 1151–1160.

[ref28] Kazai K. Yagi A. (2003). Comparison between the lambda response of eye-fixation-related potentials and the P100 component of pattern-reversal visual evoked potentials. Cogn. Affect. Behav. Neurosci. 3, 46–56. doi: 10.3758/CABN.3.1.46, PMID: 12822598

[ref29] Laird J. D. Lacasse K. (2014). Bodily influences on emotional feelings: accumulating evidence and extensions of William James’s theory of emotion. Emot. Rev. 6, 27–34. doi: 10.1177/1754073913494899

[ref30] Lazarus R. Folkman S. (1984). Stress, appraisal, and coping. Cham: Springer Publishing.

[ref31] Lee C. Miyakoshi M. Delorme A. Cauwenberghs G. Makeig S. (2015). Non-parametric group-level statistics for source-resolved ERP analysis. Eng. Med. Biol. Soc. 2015, 7450–7453. doi: 10.1109/EMBC.2015.7320114, PMID: 26738014 PMC6620019

[ref32] Lehmann D. (1987). Principles of spatial analysis. Handb. Electroencephal. Clin. Neurophysiol. 1, 309–354.

[ref33] Lipnicki D. M. Byrne D. G. (2005). Thinking on your back: solving anagrams faster when supine than when standing. Cogn. Brain Res. 24, 719–722. doi: 10.1016/j.cogbrainres.2005.03.003, PMID: 16099373

[ref34] Lipnicki D. M. Byrne D. G. (2008). An effect of posture on anticipatory anxiety. Int. J. Neurosci. 118, 227–237. doi: 10.1080/00207450701750463, PMID: 18205079

[ref35] Luck S. J. Woodman G. F. Vogel E. K. (2000). Event-related potential studies of attention. Trends Cogn. Sci. 4, 432–440. doi: 10.1016/S1364-6613(00)01545-X 11058821

[ref36] Lundqvist D. Flykt A. Öhman A. (1998). The Karolinska directed emotional faces (KDEF). CD ROM from Department of Clinical Neuroscience. Psychol. Sec. 91:630.

[ref37] Lundström J. N. Boyle J. A. Jones-Gotman M. (2006). Sit up and smell the roses better: olfactory sensitivity to phenyl ethyl alcohol is dependent on body position. Chem. Senses 31, 249–252. doi: 10.1093/chemse/bjj025, PMID: 16394243

[ref38] Makeig S. Bell A. J. Jung T.-P. Sejnowski T. J. (1996). Independent component analysis of electroencephalographic data. Adv. Neural Inf. Proces. Syst. 3, 145–151.

[ref39] Maris E. Oostenveld R. (2007). Nonparametric statistical testing of EEG-and MEG-data. J. Neurosci. Methods 164, 177–190. doi: 10.1016/j.jneumeth.2007.03.024, PMID: 17517438

[ref40] Mazaheri A. Picton T. W. (2005). EEG spectral dynamics during discrimination of auditory and visual targets. Cogn. Brain Res. 24, 81–96. doi: 10.1016/j.cogbrainres.2004.12.013, PMID: 15922161

[ref41] Mergner T. Rosemeier T. (1998). Interaction of vestibular, somatosensory and visual signals for postural control and motion perception under terrestrial and microgravity conditions - a conceptual model. Brain Res. Rev. 28, 118–135. doi: 10.1016/S0165-0173(98)00032-0, PMID: 9795180

[ref42] Nashner L. M. (1976). Adapting reflexes controlling the human posture. Exp. Brain Res. 26, 59–72. doi: 10.1007/BF00235249 964327

[ref43] Oostenveld R. Oostendorp T. F. (2002). Validating the boundary element method for forward and inverse EEG computations in the presence of a hole in the skull. Hum. Brain Mapp. 17, 179–192. doi: 10.1002/hbm.10061 12391571 PMC6872070

[ref44] Ouchi Y. Okada H. Yoshikawa E. Futatsubashi M. Nobezawa S. (2001). Absolute changes in regional cerebral blood flow in association with upright posture in humans: an orthostatic PET study. J. Nuc. Med. Off. Publ. Med. 42, 707–712, PMID: 11337564

[ref45] Ouchi Y. Okada H. Yoshikawa E. Nobezawa S. Futatsubashi M. (1999). Brain activation during maintenance of standing posture in humans. Brain 122, 329–338. doi: 10.1093/brain/122.2.329 10071060

[ref46] Pourtois G. Dan E. S. Grandjean D. Sander D. Vuilleumier P. (2005). Enhanced extrastriate visual response to bandpass spatial frequency filtered fearful faces: time course and topographic evoked-potentials mapping. Hum. Brain Mapp. 26, 65–79. doi: 10.1002/hbm.20130, PMID: 15954123 PMC6871777

[ref47] Price T. F. Dieckman L. W. Harmon-Jones E. (2012). Embodying approach motivation: body posture influences startle eyeblink and event-related potential responses to appetitive stimuli. Biol. Psychol. 90, 211–217. doi: 10.1016/j.biopsycho.2012.04.001, PMID: 22522185

[ref48] Protzak J. Gramann K. (2018). Investigating established EEG parameter during real-world driving. Front. Psychol. 9:275396. doi: 10.3389/fpsyg.2018.02289, PMID: 30532722 PMC6265363

[ref49] Quiroga R. Q. Rosso O. A. Basar E. Schurmann M. (2001). Wavelet entropy in event-related potentials: a new method shows ordering of EEG oscillations. Biol. Cybern. 84, 291–299. doi: 10.1007/s004220000212, PMID: 11324340

[ref50] Raz A. Lieber B. Soliman F. Buhle J. Posner J. Peterson B. S.. (2005). Ecological nuances in functional magnetic resonance imaging (fMRI): psychological stressors, posture, and hydrostatics. Neuro Image 25, 1–7. doi: 10.1016/j.neuroimage.2004.11.015, PMID: 15734338

[ref51] Rice J. K. Rorden C. Little J. S. Parra L. C. (2013). Subject position affects EEG magnitudes. Neuro Image 64, 476–484. doi: 10.1016/j.neuroimage.2012.09.041, PMID: 23006805

[ref52] Riskind J. H. Gotay C. C. (1982). Physical posture: could it have regulatory PR feedback effects on motivation and Emoton? Motiv. Emot. 6, 273–298. doi: 10.1007/BF00992249

[ref53] Roberts H. Soto V. Tyson-Carr J. Kokmotou K. Cook S. Fallon N.. (2018). Tracking economic value of products in natural settings: a wireless EEG study. Front. Neurosci. 12:910. doi: 10.3389/fnins.2018.00910, PMID: 30618548 PMC6306680

[ref54] Schweizer T. A. Kan K. Hung Y. Tam F. Naglie G. Graham S. J. (2013). Brain activity during driving with distraction: an immersive fMRI study. Front. Hum. Neurosci. 7:53. doi: 10.3389/fnhum.2013.00053 23450757 PMC3584251

[ref55] Soto V. Tyson-Carr J. Kokmotou K. Roberts H. Cook S. Fallon N.. (2018). Brain responses to emotional faces in natural settings: a wireless Mobile EEG recording study. Front. Psychol. 9:2003. doi: 10.3389/fpsyg.2018.02003, PMID: 30410458 PMC6209651

[ref56] Sun C. Harmon-Jones E. (2021). Supine body posture reduces cognitive conflict processing: evidence from N450 Stroop interference. Psychophysiology 58:e13693. doi: 10.1111/psyp.13693, PMID: 32996615

[ref57] Świrski L. Bulling A. Dodgson N. Swirski L. Bulling A. Dodgson N. (2012). “Robust real-time pupil tracking in highly off-axis images,” in *Proceedings of the Symposium on Eye Tracking Research and Applications-ETRA’12*, 173.

[ref58] The MathWorks Inc. (2018). MATLAB version: 9.5.0 (R2018b). Natick, MA: The MathWorks Inc.

[ref59] Thibault R. T. Lifshitz M. Jones J. M. Raz A. (2014). Posture alters human resting-state. Cortex 58, 199–205. doi: 10.1016/j.cortex.2014.06.014 25041937

[ref60] Thibault R. T. Raz A. (2016). Imaging Posture Veils Neural Signals. Front. Hum. Neurosci. 10, 1–8. doi: 10.3389/fnhum.2016.00520, PMID: 27818629 PMC5073137

[ref61] Thickbroom G. W. Knezevič W. Carroll W. M. Mastaglia F. L. (1991). Saccade onset and offset lambda waves: relation to pattern movement visually evoked potentials. Brain Res. 551, 150–156. doi: 10.1016/0006-8993(91)90927-N, PMID: 1913148

[ref62] Torriente I. Valdes-Sosa M. Ramirez D. Bobes M. A. (1999). Visual evoked potentials related to motion-onset are modulated by attention. Vis. Res. 39, 4122–4139. doi: 10.1016/S0042-6989(99)00113-3, PMID: 10748944

[ref63] Tottenham N. Tanaka J. W. Leon A. C. McCarry T. Nurse M. Hare T. A.. (2009). The NimStim set of facial expressions: judgements from untrained research participants. Psychiatry Res. 168, 242–249. doi: 10.1016/j.psychres.2008.05.006, PMID: 19564050 PMC3474329

[ref64] Troncoso A. Blanco K. Rivera-Rei Á. Martínez-Pernía D. (2024). Empathy bodyssence: temporal dynamics of sensorimotor and physiological responses and the subjective experience in synchrony with the other’s suffering. Front. Psychol. 15:1362064. doi: 10.3389/fpsyg.2024.1362064, PMID: 38577111 PMC10994162

[ref65] Van Voorhis S. Hillyard S. A. (1977). Visual evoked potentials and selective attention to points in space. Percept. Psychophys. 22, 54–62. doi: 10.3758/BF03206080

[ref66] Vigon L. Saatchi R. Mayhew J. E. W. Taroyan N. A. Frisby J. P. (2002). Effect of signal length on the performance of independent component analysis when extracting the lambda wave. Med. Biol. Eng. Comput. 40, 260–268. doi: 10.1007/BF02348134, PMID: 12043810

[ref67] Yagi A. (1981). Visual signal detection and lambda responses. Electroencephalogr. Clin. Neurophysiol. 52, 604–610. doi: 10.1016/0013-4694(81)91434-6 6172259

[ref68] Zink R. Hunyadi B. Van Huffel S. Vos M. (2016). Mobile EEG on the bike: disentangling attentional and physical contributions to auditory attention tasks. J. Neural Eng. 13:046017. doi: 10.1088/1741-2560/13/4/046017, PMID: 27351459

